# Synergistic chemo-photothermal therapy using gold nanorods supported on thiol-functionalized mesoporous silica for lung cancer treatment

**DOI:** 10.1038/s41598-024-54778-3

**Published:** 2024-02-22

**Authors:** Maryam Deinavizadeh, Ali Reza Kiasat, Mohammad Shafiei, Mohammad Sabaeian, Roya Mirzajani, Seyed Mohammadsaleh Zahraei, Fateme Khalili, Minmin Shao, Aimin Wu, Pooyan Makvandi, Nasrin Hooshmand

**Affiliations:** 1 Department of Chemistry, Faculty of Science, Shahid Chamran University of Ahvaz, Ahvaz, Iran; 2 Petroleum Geology and Geochemistry Research Center (PGGRC), Shahid Chamran University of Ahvaz, Ahvaz, Iran; 3 Department of Biology, Faculty of Science, Shahid Chamran University of Ahvaz, Ahvaz, Iran; 4 Department of Physics, Faculty of Science, Shahid Chamran University of Ahvaz, Ahvaz, Iran; 5 Center for Research On Laser and Plasma, Shahid Chamran University of Ahvaz, Ahvaz, Iran; 6 Department of Otorhinolaryngology, The Second Affiliated Hospital of Shanghai University, Wenzhou Central Hospital, Wenzhou, China; 7 Department of Orthopaedics, Key Laboratory of Structural Malformations in Children of Zhejiang Province, Key Laboratory of Orthopaedics of Zhejiang Province, The Second Affiliated Hospital and Yuying Children’s Hospital of Wenzhou Medical University, Wenzhou, 325000 Zhejiang China; 8 Centre of Research Impact and Outcome, Chitkara University, Rajpura, 140401 Punjab India; 9 Department of Biomaterials, Saveetha Dental College and Hospitals, SIMATS, Saveetha University, Saveetha University, Chennai, 600077 India; 10 Laser Dynamics Laboratory, School of Chemistry and Biochemistry, Georgia Institute of Technology, Atlanta, GA 30332 USA

**Keywords:** Chemo-photothermal therapy, Chemotherapy, Photothermal therapy, Gold Nanorods, Cancer Cells, Doxorubicin, Dual-responsive, Lung cancer cells, Mesoporous Silica MCM-41, Biochemistry, Biotechnology, Cancer, Chemistry, Nanoscience and technology, Optics and photonics

## Abstract

Cancer therapy necessitates the development of novel and effective treatment modalities to combat the complexity of this disease. In this project, we propose a synergistic approach by combining chemo-photothermal treatment using gold nanorods (AuNRs) supported on thiol-functionalized mesoporous silica, offering a promising solution for enhanced lung cancer therapy. To begin, mesoporous MCM-41 was synthesized using a surfactant-templated sol–gel method, chosen for its desirable porous structure, excellent biocompatibility, and non-toxic properties. Further, thiol-functionalized MCM-41 was achieved through a simple grafting process, enabling the subsequent synthesis of AuNRs supported on thiol-functionalized MCM-41 (AuNR@S-MCM-41) via a gold-thiol interaction. The nanocomposite was then loaded with the anticancer drug doxorubicin (DOX), resulting in AuNR@S-MCM-41-DOX. Remarkably, the nanocomposite exhibited pH/NIR dual-responsive drug release behaviors, facilitating targeted drug delivery. In addition, it demonstrated exceptional biocompatibility and efficient internalization into A549 lung cancer cells. Notably, the combined photothermal-chemo therapy by AuNR@S-MCM-41-DOX exhibited superior efficacy in killing cancer cells compared to single chemo- or photothermal therapies. This study showcases the potential of the AuNR@S-MCM-41-DOX nanocomposite as a promising candidate for combined chemo-photothermal therapy in lung cancer treatment. The innovative integration of gold nanorods, thiol-functionalized mesoporous silica, and pH/NIR dual-responsive drug release provides a comprehensive and effective therapeutic approach for improved outcomes in lung cancer therapy. Future advancements based on this strategy hold promise for addressing the challenges posed by cancer and transforming patient care.

## Introduction

Cancer, a complex and devastating disease, requires innovative therapeutic strategies that can effectively target tumor cells while minimizing adverse effects on healthy tissues^1–3^. Photothermal therapy (PTT) is a highly selective and noninvasive therapeutic technique that has shown great promise for the treatment of cancer^4^. Nowadays, various nanoscale materials have been widely explored as photothermal agents (PTAs).

Among these nanomaterials, gold-based nanoarchitectures (e.g., nanorods, nanostars, nanocages, nanospheres and nanoshells) have been extensively studied as PTAs due to their tunable localized surface plasmon resonance (LSPR) properties^5–7^, low toxicity and excellent biocompatibility^8,9^. Gold nanorods (GNRs) are particularly interesting for concurrent cancer therapy due to their unique properties such as their excellent biocompatibility, controllable size, tunable surface plasmon resonance (SPR)^10^, high photothermal conversion efficiency and ease of surface modification^11–13^. However, AuNRs can aggregate in the tumor microenvironment which reduces their optical attributes and diminishes the photothermal therapy efficacy^14^. Thus, surface modification of AuNRs by non-toxic and biocompatible stabilizers is necessary to improve their stability and PTT efficacy^15–19^.

Photothermal therapy using photothermal agents alone has limitations in completely destroying cancer cells^20^. Light penetration into deeper tumor tissues is reduced due to absorption and scattering, leading to decreased photothermal therapy efficiency^21^. Researchers have combined PTT with additional therapeutic strategies to dominate these limitations and improve effectiveness^22–26^. Among various combination techniques, co-delivering anticancer drugs with photothermal agents has emerged as a highly promising approach. In this combination photothermal chemotherapy, the photothermal agent enables localized heating while the anticancer drug provides cytotoxic effects^27–31^. Various materials were conjugated to gold nanorods (AuNRs) to enable drug loading for combination photothermal chemotherapy, including cell membrane coatings. These materials can target cancer cells specifically, deliver therapeutic agents to the tumor location, and induce hyperthermia to increase the therapeutic effect^32,33^.

Mesoporous silica nanoparticles feature a variety of advantages, including simple surface modification, good biocompatibility and great chemical and mechanical stability^34^.

Among the family of mesoporous silica nanoparticles, MCM-41 (Mobil Composition of Matter No. 41) possesses promising characteristics, instance e.g., having hexagonally shaped pores, an ordered mesoporous structure, enormous surface area (about 900–1500 m^2^ g^−1^) along with a narrow pore size distribution (2–10 nm)^35–37^. Furthermore, the presence of a large number of silanol groups (Si–OH) on the surface MCM-41 simplifies functionalization of MCM-41, and the pore size can be appropriate for encapsulation of bioactive molecules^38–41^. Furthermore, synergetic pH/NIR responsive drug delivery systems have been shown to be a promising strategy for selectively delivering anticancer drugs in tumors. These systems improve permeability and uptake of the targeted cells, as a result ameliorate the effectiveness of chemotherapy. Besides, these nanoscale materials can generate the hyperthermia under NIR laser to destroy tumor cells; this offers combined photothermal and chemotherapy^42–45^.

By keeping these facts in mind and in continuing our research on the development of pH/NIR dual-responsive drug delivery using AuNRs@DOX for combined chemo-photothermal therapy^46–48^, in this work, we developed AuNRs supported on thiol-MCM-41 (AuNR@S-MCM-41) as a drug support and designed the AuNR@S-MCM-41-DOX nanocomposite for controlled release of bioactive molecules and synergetic photothermal and chemotherapy techniques. First, gold nanorods were supported on thiolated MCM-41 via Au–S bonds (AuNR@S-MCM-41). Then, the anticancer drug doxorubicin (DOX) was loaded into the AuNR@S-MCM-41 nanocomposite by strong electrostatic interactions. Under near-infrared irradiation, we expected the AuNR@S-MCM-41-DOX nanocomposite to exhibit pH/NIR dual-responsive drug release behaviors and improve the efficacy of chemo-photothermal therapy compared to single therapy. The results of in vitro experiments conducted in this study showed that AuNR@S-MCM-41-DOX is both biocompatible and effective against A549 lung cancer cells. Overall, the nanocomposite demonstrated stimuli-responsive drug delivery performance, in which release is facilitated by internal and external triggers (e.g., tumor microenvironment and NIR laser).

## Experimental section

### Generals

Cetyltrimethylammonium bromide (CTAB), sodium borohydride (NaBH_4_), L-ascorbic acid, Silver Nitrate (AgNO_3_), Gold (III) chloride solution (HAuCl_4_.3H_2_O), [3-(4,5- dimethylthiazol-2-yl)-3,5-diphenytetrazolium bromide] (MTT), tetraethylorthosilicate (TEOS), (3-mercatopropyl)trimethoxy-silane (MPTMS), sodium hydroxide (NaOH), ethanol (EtOH), toluene, and hydrochloric acid (HCl) were purchased from Sigma-Aldrich. Doxorubicin hydrochloride (DOX) as an anti-cancer drug was obtained from Fenghua Lianbo. Co (Peking, China). Deionized water was used in all the experiments. Human lung cancer cell line A549 was obtained from the Pasteur Institute of Iran.

The absorbance of the AuNRs was measured using a UV–Vis spectrophotometer (TU-1901). To determine the size of the nanoparticles, a transmission electron microscope (TEM) (LEO-906E-80 kV) was used. Fourier transform infrared (FTIR) spectra were recorded using BOMEM MB-Series 1998 FT-IR spectrometer. To characterize the surface of the nanoparticles, a Zeta sizer Nano ZS instrument (Malvern Instruments, U.K.) was used to measure the surface zeta potentials. The An ICP-OES spectrometer (Optima 8300, Perkin Elmer) was used for determination of Au concentration in samples. Energy-dispersive X-ray spectroscopy (EDX) and EDX elemental mapping analysis were performed by FEI Tecnai G2 F20S-TWIN at 200 kV. A continuous wave laser with wavelength 808 nm (GCSLS-05-7W00 fiber-coupled, Daheng Science and Technology, China) was used for the laser irradiation experiment (7 mm, 1.4 W, 3.6 W/cm^2^).

### Photothermal performance

To evaluate the photothermal effect of the AuNR@S-MCM-41 nanocomposite, 100 µL of nanocomposite dispersions at various concentrations (0–25 nM) were added to a 96-well plate along with control wells. The control sample was contained 100 µL of medium (DMEM) without any nanoparticle. Other wells contain nanoparticles with different concentrations. The plate was irradiated with a diode laser at 808 nm and a beam diameter of 7 mm, using a power density of 3.6 W/cm^2^ for 5 min. The temperature of the solutions was measured using a thermocouple during laser irradiation.

### Preparation of AuNR@S-MCM-41-DOX

A solution of DOX at a concentration of 5 µM (1 mL) was added to solution of AuNR@S-MCM-41 nanocomposite (5 mg, 5 mL) at a concentration of 228 nM in PBS buffer and stirred for 24 h at room temperature. The resulting AuNR@S-MCM-41-DOX was then separated and washed through centrifugation at 11,000 rpm for 10 min. The concentration of DOX in the supernatant solution was measured by a UV–Vis spectrometer at 485 nm. The efficiency percentages of DOX loading and entrapment were calculated using Eqs. (1) and (2), respectively.

1 $$DLE\left(\%\right)= \frac{(Total\,amount\,of\,DOX)-(Residual\,amount\,of\,DOX)}{(Total\,amount\,of\,Nanocomposite)}\times 100$$

2 $$EE\left(\%\right)= \frac{{Abs}_{\left(original\,DOX\right)}-{Abs}_{\left(residual\,DOX\right)}}{{Abs}_{\left(original\,DOX\right)}}\times 100$$

### In vitro drug release performance of AuNR@S-MCM-41-DOX

In this experiment, a 5 mL dispersion of AuNR@S-MCM-41-DOX in PBS buffer was agitated at 130 rpm and 37 °C in the dark, with DOX concentration of 5 µM, at pH values of 5.5 or 7.4. After centrifuging the dispersions at various time intervals, the amount of released DOX in the supernatant was evaluated using a UV–Vis spectrometer at 485 nm. Additionally, DOX release was assessed under 808 nm laser irradiation (power density of 3.6 W cm^−2^) in PBS with pH values of 5.5 and 7.4.

## Results and discussion

### Synthesis and characterization of AuNR@S-MCM-41-DOX

**Scheme 1:**
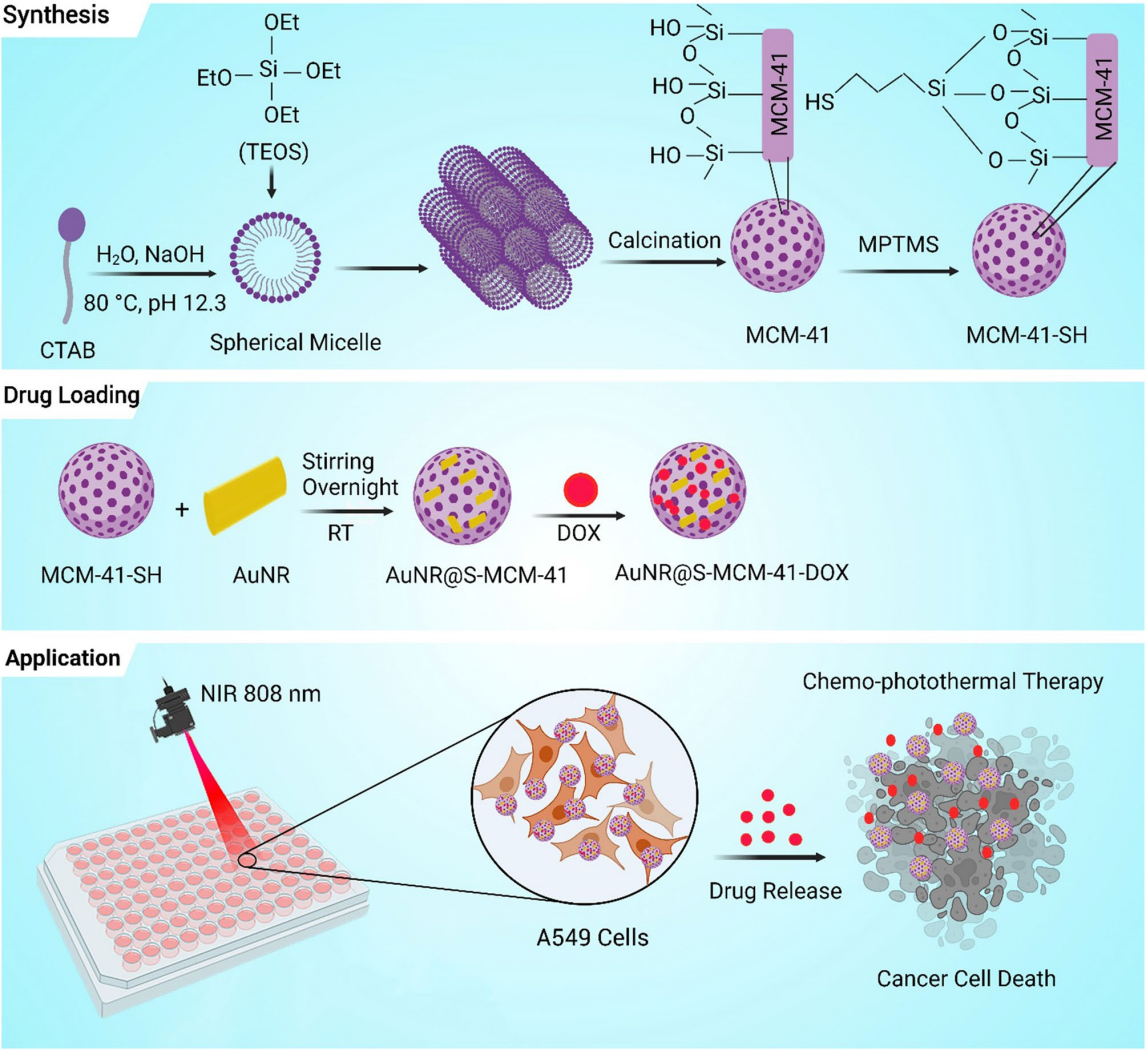
Schematic illustration for the synthesis of AuNR@S-MCM-41-DOX nanocomposite. CTAB: Cetyltrimethyl ammonium bromide, TEOS: Tetraethyl orthosilicate, MPTMS: (3-Mercaptopropyl)trimethoxysilane, DOX: Doxorubicin.

The preparation steps of AuNR@S-MCM-41-DOX nanocomposite as summarized in Scheme 1 are:

- A: synthesis of MCM-41 nanoparticles by surfactant-templated sol–gel method
- B: synthesis of MCM-41-SH via post grafting method using 3-mercaptopropyl trimethoxysilane
- C: preparation of AuNRs through seed-mediated growth method and assembly of MCM-41-SH on AuNRs via a gold–thiol interaction
- D: loading of DOX on AuNR@S-MCM-41 nanocomposite by strong electrostatic interactions

**Figure 1:**
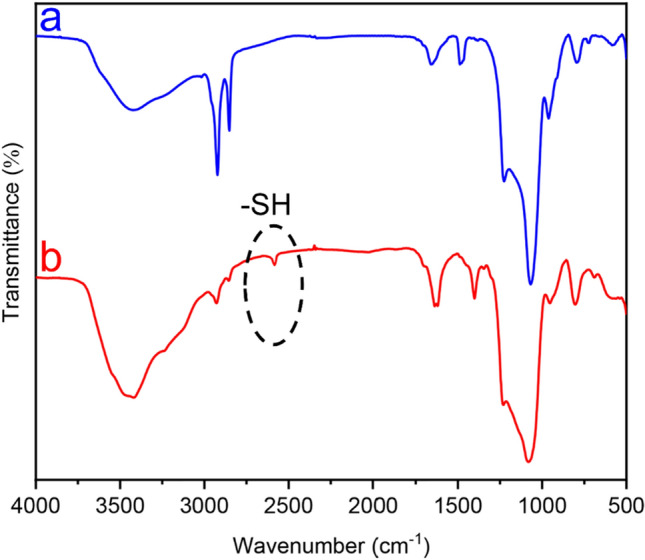
FTIR spectra of (**a**) MCM-41 and (**b**) MCM-41-SH.

The successful synthesis of MCM-41-SH nanoparticles was confirmed by the FTIR spectra. The FTIR spectra of MCM-41 and MCM-41-SH are indicated in Fig. 1. The FTIR spectrum of MCM-41-SH (Fig. 1b) compared to that of MCM-41 (Fig. 1a) indicated the presence of the S–H stretching vibration in MCM-41-SH, which was confirmed by the detection of a weak vibrational band at 2583 cm^−1^. This qualitative confirmation from the FTIR spectra displays that the thiol functional groups were successfully included into the MCM-41 nanoparticles^49^.

**Figure 2:**
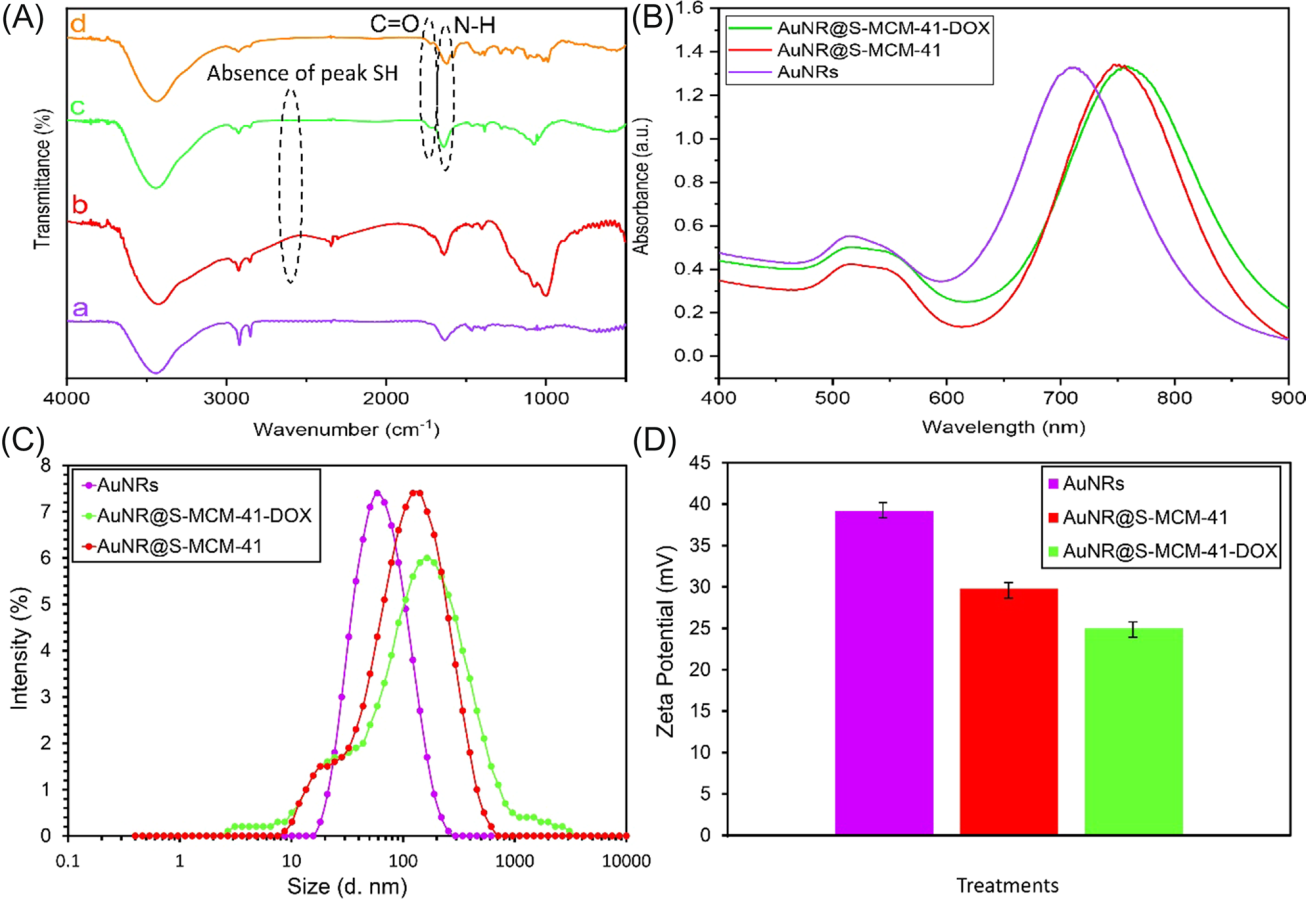
(**A**) FTIR spectra of AuNRs (**a**), AuNR@S-MCM-41 (**b**), AuNR@S-MCM-41-DOX (**c**), DOX (**d**); (**B**) the UV–Vis absorption spectra of AuNRs, AuNR@S-MCM-41 and AuNR@S-MCM-41-DOX; Hydrodynamic size analysis by DLS (**C**) and Surface zeta potential (**D**) of AuNRs, AuNR@S-MCM-41, and AuNR@S-MCM-41-DOX.

The successful synthesis of AuNRs, AuNR@S-MCM-41, AuNR@S-MCM-41-DOX, and DOX was confirmed by FTIR spectra (Fig. 2A). The disappearance of the -SH absorption peak at 2583 cm^−1^ in AuNR@S-MCM-41 and AuNR@S-MCM-41-DOX confirms the formation of gold-sulfur covalent bonds. The FT-IR spectrum of DOX indicates two peaks at 1584 cm^−1^ and 1622 cm^−1^ (N–H) and one at 1725 cm^−1^ (C = O), which are also present but relatively weak in the FTIR spectrum of AuNR@S-MCM-41-DOX nanocomposite, confirming drug loading.

Also, the successful coating of AuNRs with MCM-41-SH and doxorubicin loading were verified through UV–Vis spectroscopy (Fig. 2B). AuNRs indicated transverse and longitudinal bands around 517 and 710 nm, respectively. The LSPR band was red shifted to nearly 748 nm on surface coating with MCM-41-SH, demonstrating the change in electronic conjugation following the chemical reaction and the Au–S bond formation, although the plasmon transverse band (519 nm) stayed approximately unchanged. Finally, successful loading of DOX in AuNR@S-MCM-41 was indicated by an LSPR to 760 nm.

The structure of AuNRs, AuNR@S-MCM-41, and AuNR@S-MCM-41-DOX were also analyzed by dynamic light scattering (DLS). The DLS measurements indicated that AuNRs, AuNR@S-MCM-41, and AuNR@S-MCM-41-DOX had hydrodynamic sizes about 54, 122.4 and 164.2 nm, respectively (Fig. 2C). The effect of hydrogen bonding between the amine and hydroxyl groups of DOX and the silanol hydroxyl groups of MCM-41 in the AuNRs@S-MCM-41-DOX nanocomposite, which produces aggregation, is the most likely the reason for the potential enhancement in the size of nanoparticles following loading of DOX.

The zeta potential of AuNRs in water (+ 39.2 mV) is likely the result of the presence of positively charged groups (CTAB) on the surface of the gold nanorods. When MCM-41-SH, which contains thiol groups, was used as a support for AuNRs, the zeta potential decreased to + 29.8 mV. This decrease in zeta potential is probably due to the introduction of negatively charged functional groups (thiol). DOX loading onto the AuNR@S-MCM-41 nanocomposite further reduces the zeta potential to + 25 mV. This decrease in zeta potential can be related to the interaction between the positively charged DOX molecules and the negatively charged functional groups on the surface of the nanocomposite, which leads to a decrease in the overall positive charge and thus a decrease in zeta potential (Fig. 2D).

**Figure 3:**
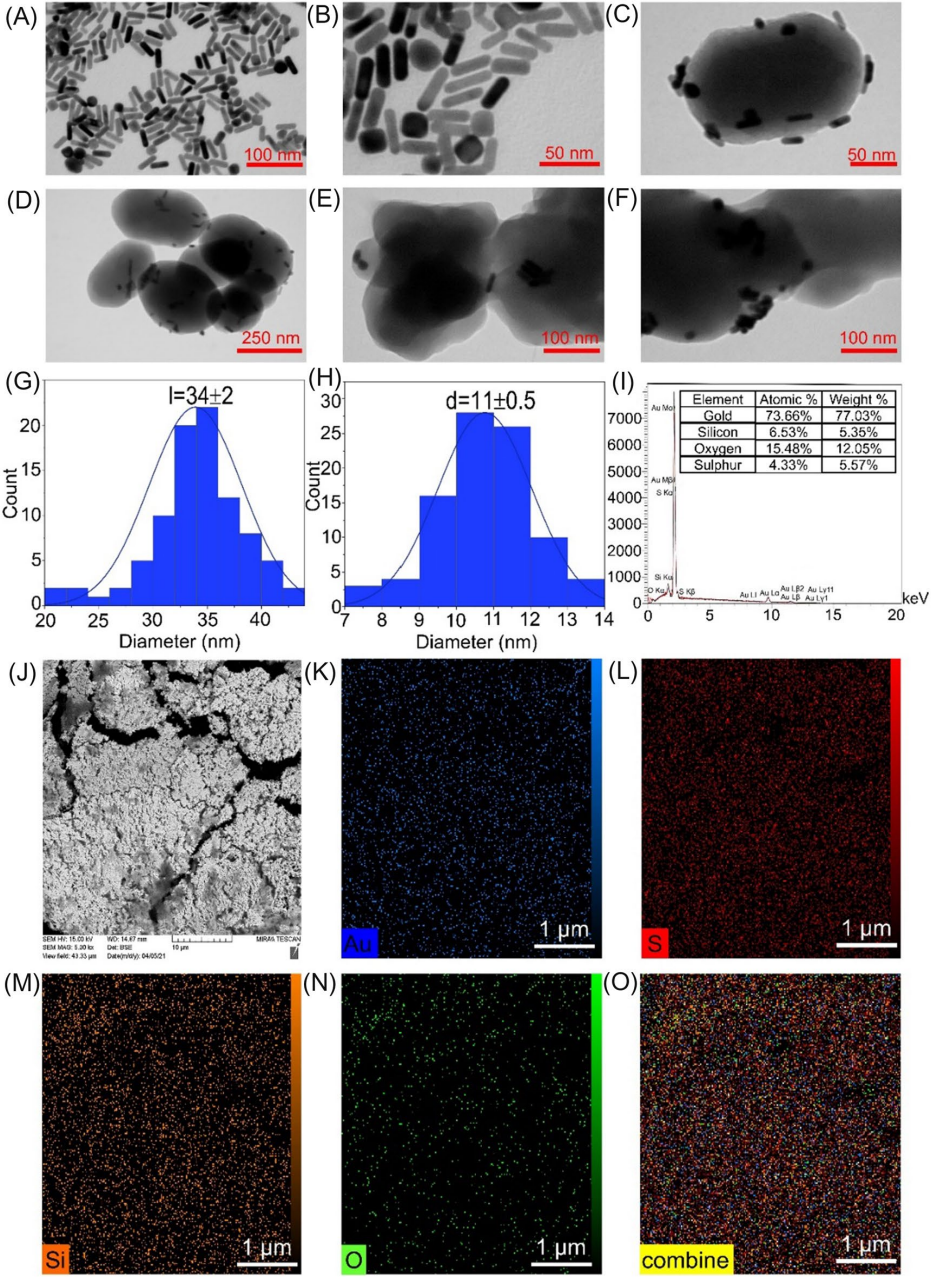
TEM images of (**A**), (**B**) AuNRs, (**C**), (**D**) AuNR@S-MCM-41, (**E**), (**F**) AuNR@S-MCM-41-DOX; (**G**), (**H**) Statistical data of length and diameter of more than 100 AuNRs as shown in (**A**), (**B**); (**I**) EDX and (**J**–**O**) SEM and EDX maps of AuNR@S-MCM-41, Au, S, Si, O and combined.

After synthesis and characterization of MCM-41-SH, gold nanorods were achieved by the seed-mediated growth method and their morphology was confirmed by TEM (Fig. 3A,B). The TEM images of more than 100 AuNRs were examined, and Image J software revealed that the average width and length of AuNRs were 11 ± 0.5 nm and 34 ± 2 nm, respectively, with an aspect ratio of 3.1 (Fig. 3G,H). Using ICP-OES, the concentration of Au atoms in AuNRs was calculated. Then, MCM-41-SH nanoparticles were applied as uniform and water-dispersible mesoporous structures as AuNR nanocarriers. The successfully prepared AuNR@S-MCM-41 nanocomposite was imaged by TEM (Fig. 3C,D). Finally, for the loading and delivery of the anticancer drug doxorubicin (DOX), AuNR@S-MCM-41 nanocomposite was utilized as a nanocarrier. The TEM images of the AuNR@S-MCM-41-DOX nanocomposite (Fig. 3E,F) confirm the loading of DOX in the AuNR@S-MCM-41 nanocomposite. The spatial distribution of Au, S, Si, and O on AuNR@S-MCM-41 was revealed via energy-dispersive X-ray spectroscopy (EDX) and elemental mapping further confirming that AuNR@S-MCM-41 nanocomposite was achieved (Fig. 3I–O).

### Photothermal properties of AuNR@S-MCM-41

**Figure 4:**
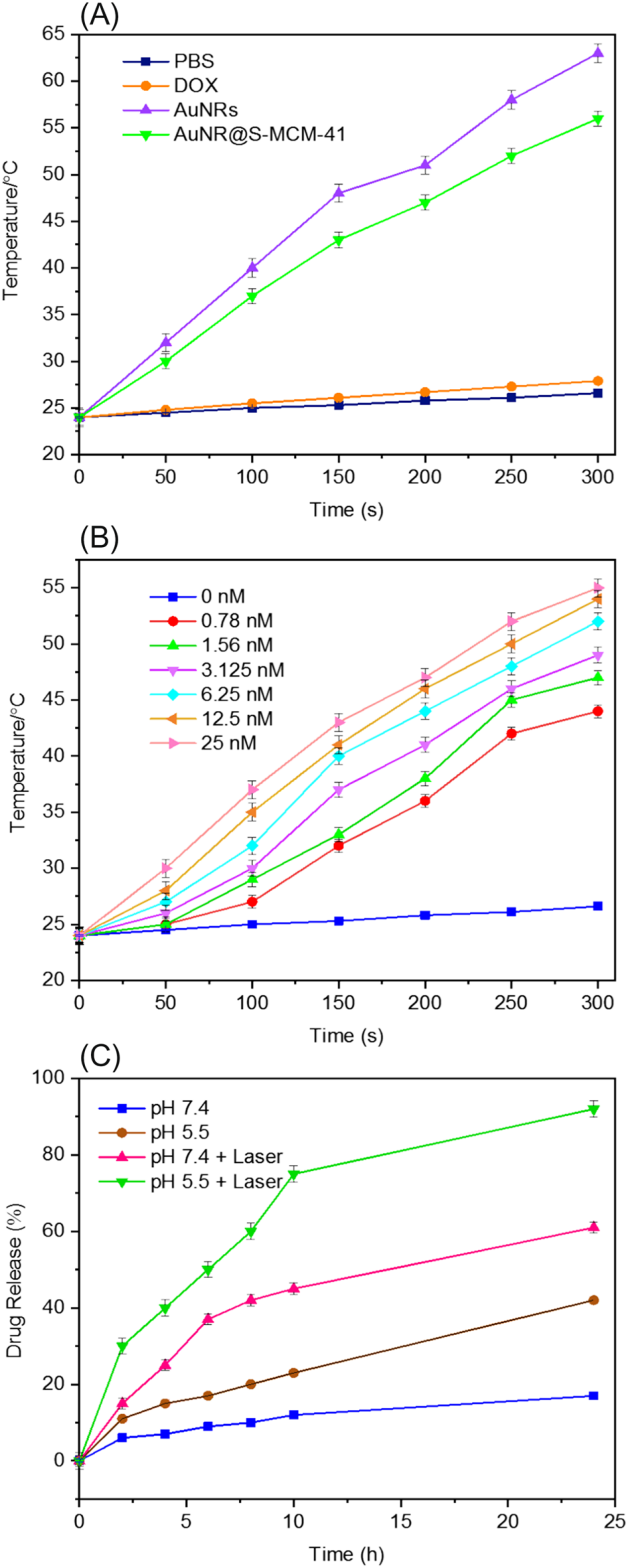
(**A**) Temperature elevation of PBS, DOX, AuNRs, and AuNR@S-MCM-41 (25 nM AuNR) after irradiating with 808 nm laser intensity (3.6 W cm^−2^); (**B**) Temperature evolution curves of the solutions containing various concentrations of AuNR@S-MCM-41 (0, 0.78, 1.56, 3.125, 6.25, 12.5 and 25 nM) under NIR laser irradiation with power density of 3.6 W cm^−2^. (**C**) Cumulative DOX release from AuNR@S-MCM-41-DOX in PBS at pH 7.4 and 5.5 without and with NIR irradiation.

Near-infrared (NIR) light is a favorable source of light in photothermal therapy as it is transparent to biological systems and has sufficient tissue penetration depth. This can increase the photothermal (PT) efficiency of cancer cells by converting incident radiation into heat and reduce the side effects on normal cells^50^. The photothermal properties of the AuNR@S-MCM-41 nanocomposite were studied by monitoring the temperature enhancement of the nanocomposite suspensions under 808 nm diode laser irradiation with a power density of 3.6 W/cm^2^. The suspensions of AuNRs, AuNR@S-MCM-41 (with an equal concentration of 25 nM of AuNRs), and solutions of PBS and DOX were irradiated using the 808 laser. As displayed in Fig. 4A, the temperature of the AuNRs and AuNRs@S-MCM-41 suspension increased from 24 to 63 °C and 24 to 56 °C after 5 min of irradiation, respectively. Meanwhile, the temperature of the PBS and DOX solutions did not significantly increase under the same conditions. These findings demonstrate that the AuNR@S-MCM-41 nanocomposite has remarkable potential for effectively converting light energy to heat energy. Next, the photothermal efficacy of the nanocomposite was evaluated at various concentrations ranging from 0 to 25 nM using NIR laser irradiation (power density of 3.6 W/cm^2^) (Fig. 4B). The results exhibited a dose-dependent behavior, where the temperature increased with increasing concentrations of AuNRs@S-MCM-41 nanocomposite. Overall, the excellent efficiency of this nanosystem in converting light energy to heat suggests that it can be potentially used as a promising candidate for cancer photothermal therapy.

### Anticancer drug loading, entrapment efficiency and release performance

DOX was easily loaded in the AuNR@S-MCM-41 nanocomposite in the phosphate-buffered saline (PBS) solution due to the nanocomposite being mesoporous, and its loading and entrapment efficiency were 26.36 and 48.27 wt%, respectively. As shown in Fig. 4C, the cumulative drug release from the AuNR@S-MCM-41-DOX was studied at pH 7.4 or 5.5 with and without 808 nm laser irradiation. The amount of DOX release from the AuNR@S-MCM-41-DOX was influenced by the pH, leading to the amount of DOX released enhanced with time. It was also found that DOX release in the absence of laser radiation occurs faster at pH 5.5 than pH 7.4. In detail, due to the low solubility of DOX at pH 7.4, AuNR@S-MCM-41-DOX released only 17% of the total loaded DOX in the duration of 24 h. The drug release was increased within 24 h (29%) under acidic conditions at pH 5.5, which is a pH close to the late endosomes and lysosomes in the tumor cell, via protonation of silanol OH groups of AuNR@S-MCM-41-DOX; consequently, the electrostatic bond between the positively charged DOX molecules and the negatively charged of silanol OH groups of AuNR@S-MCM-41-DOX were dissociated. Due to the great solubility of DOX drug at pH 5.5, the release of it was enhanced under these conditions. The pH-triggered drug release performance under the tumor acid intracellular medium can increase the anticancer capability by liberating the drug in tumor cells^51^. Compared with our previous work^46^, the DOX release rate in AuNR@SBA-15-SH nanocomposite could reach 32% at pH 5.5. These results revealed that mesoporous silica MCM-41 were efficient in trapping loaded drugs. Also DOX loading and release in AuNR@S-MCM-41 nanocomposite were compared with AuNR@SiO_2_-TAT, which was developed using an in situ grafting-cleavage method to modify the bioactive peptide in the core–shell structure of AuNR@SiO_2_ for providing synergistic chemo-photothermal cancer treatment. The loading of DOX in AuNR@S-MCM-41 was higher than that in AuNR@SiO_2_-TAT (22.5%), and the DOX release rate in AuNR@SiO_2_-TAT was 45% at pH 5.5^52^. By contrast, the DOX release behavior of AuNR@S-MCM-41-DOX under 808 nm laser at pH 7.4 exhibited faster DOX release, with the release rate of DOX reaching 61% during 24 h. This difference was ascribed to the photothermal effects of AuNR@S-MCM-41-DOX. NIR laser irradiation provided a localized increase in temperature, which caused the dissociation of weak interactions between DOX and the silanol groups. As the pH of the released solutions reduced to 5.5, DOX release was accelerated, achieving a release rate of 92% with laser irradiation. Reducing pH may contribute to DOX release by weakening the hydrogen bonds and electrostatic binding between DOX and the carrier. Based on these results, the AuNR@S-MCM-41-DOX nanocomposite showed NIR laser and pH dual-responsive DOX drug release behaviors, increasing the efficiency of drug delivery.

### Cellular uptake

One of the key requirements for delivering anticancer drugs is the capacity to internalize into cells. ICP-OES spectrometry was used to examine the penetrating effect and consequent localization of AuNR@S-MCM-41-DOX nanocomposites in A549 lung cancer cells.

First, nanocomposites with various concentrations (0.316, 0.632, and 1.265 ppm or 0.78, 1.56, and 3.125 nM) was incubated with A549 cells for 48 h. Then the gold content endocytosed into cells was obtained using ICP-OES spectrometry (0.107, 0.31, and 0.851 ppm). The percentage of internalized Au concentration to the dose of incubated for each cell was measured to determine the cell uptake efficacy by equation (S1). The A549 cells were thoroughly washed with PBS solution before ICP-OES analysis to remove cell-adhered nanocomposites that were not internalized. Consequently, ICP analysis demonstrated the concentration of nanocomposites entering the cells. Following a 48 h incubation period, the internalized amounts of Au atoms per cell were 4.23%, 6.13%, and 8.41%, respectively, indicating that AuNR@S-MCM-41-DOX was uptaken by the cells. This confirms the cell uptake of AuNR@S-MCM-41-DOX in a concentration-dependent manner.

### In vitro combined chemo/photothermal therapy Assay

**Figure 5:**
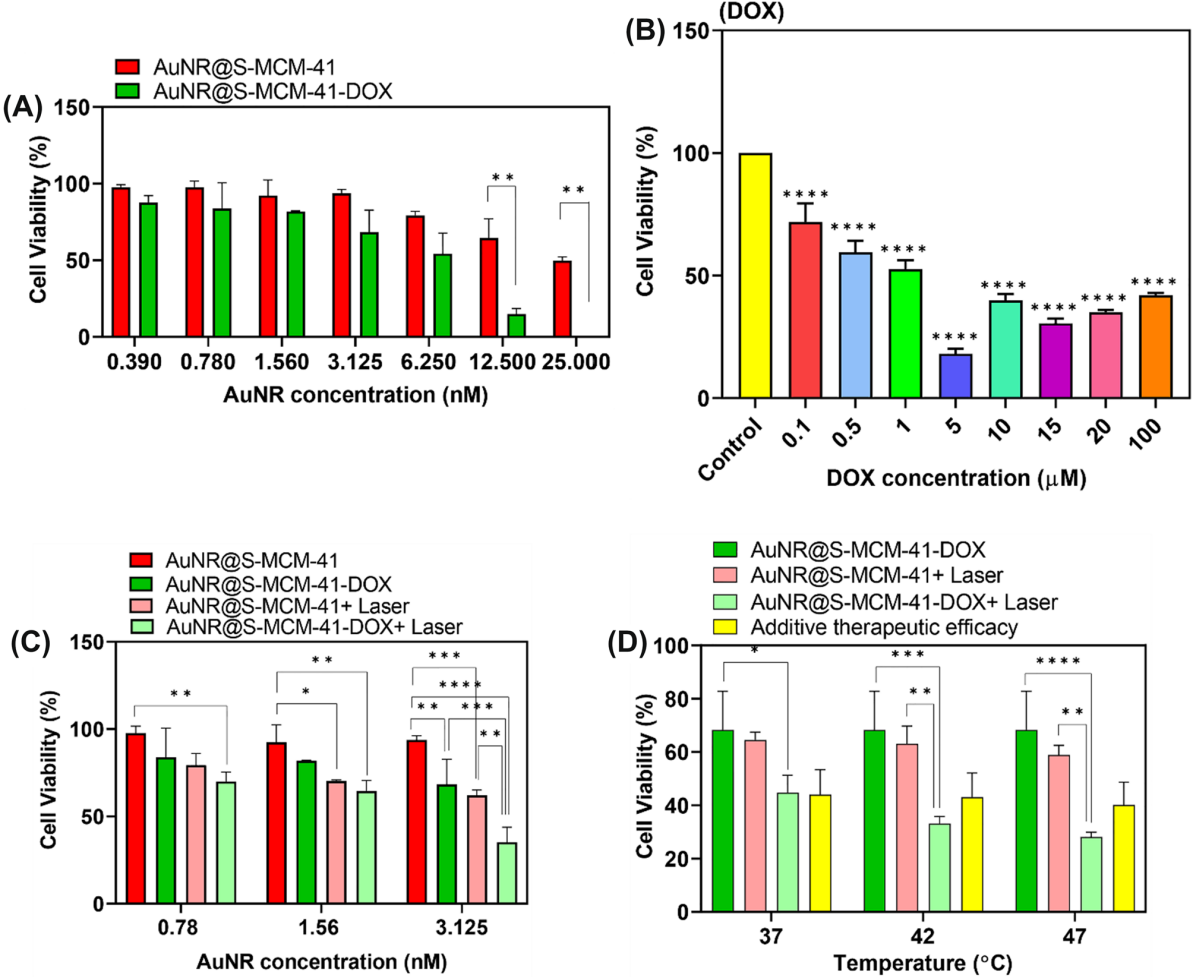
(**A**) Cell viabilities of the A549 cells after incubation with various concentrations of AuNR@S-MCM-41 and AuNR@S-MCM-41-DOX, (**B**) free DOX, (**C**) Comparative cell viabilities of A549 incubated with AuNR@S-MCM-41 or AuNR@S-MCM-41-DOX at different concentrations under exposure to 808 nm laser, (**D**) A comparison of the viability of the A549 cells treated by AuNR@S-MCM-41 + Laser, AuNR@S-MCM-41-DOX and AuNR@S-MCM-41-DOX + Laser.

To finally exhibit the near-infrared triggered photothermal-chemo therapy effect of the AuNR@S-MCM-41-DOX nanocomposite on A549 cancer cells, MTT assays were examined under various conditions. It can be seen in Fig. 5A that without irradiation of the 808 nm laser, the AuNR@S-MCM-41 nanoparticles indicated low cytotoxicity to A549 cells at different concentrations (0.39–25 nM). The cell viability was more than 82% after A549 cancer cells were incubated with various concentrations of the AuNR@S-MCM-41 nanocomposite for 48 h. The results exhibited that nanocomposite has good biocompatibility and is relatively nontoxic to cancer cells. When encapsulating DOX into AuNR@S-MCM-41, the treatment effect of AuNR@S-MCM-41-DOX can be changed significantly. The AuNR@S-MCM-41-DOX nanocomposite showed a cell viability of 56% in Fig. 5A, indicating that this nanocomposite indeed performed well in preventing the DOX from leaking. The statistically significant difference between treated cells with concentrations of 12.5 (*p* = 0.002698) and 25 (*p* = 0.000003) AuNR@S-MCM-41 and AuNR@S-MCM-41-DOX in comparison to treated with cells other concentrations were assessed with *t*-test (Fig. 5A). The results in Fig. 5B demonstrated that the viability of cells after treatment with various concentrations of free DOX was 43%. In addition, the results were analyzed and compared to the control group via one-way ANOVA (*p* ˂ 0.0001). Free DOX provided a higher IC50 than the AuNR@S-MCM-41-DOX nanocomposite (at equivalent AuNR concentrations). Therefore, after DOX was loaded in the AuNR@S-MCM-41 nanocomposite, the cell death efficacy in A549 cells through the loading DOX was enhanced (Table 1).

**Table 1:** IC50 of AuNR@S-MCM-41, AuNR@S-MCM-41-DOX and free DOX after 48 h incubation in A549 Cells.

AuNR@S-MCM-41	AuNR@S-MCM-41-DOX	Free DOX
7.46 nM	5.3 nM	1.302 µM

However, after irradiating the cells with an 808 nm laser, the cell viability of cells treated with various concentrations of AuNR@S-MCM-41 dramatically reduced. To assess the therapeutic effect of photothermal therapy, the cells were incubated for 45 h with AuNR@S-MCM-41 at concentrations (AuNRs equivalent 0.78, 1.56 and 3.125 nM). The cell medium was then heated to 37, 42, and 47 °C after 5 min of 808 nm laser irradiation, and the MTT assay was done to examine the therapeutic effect of AuNR@S-MCM-41 + Laser after incubation for 4 h. As seen in Fig. 5C, cell viabilities were negatively correlated with the concentration of the samples. The results exhibited that the AuNR@S-MCM-41 under NIR laser irradiation for 5 min with the dose of 3.125 nM may obviously cause a higher death rate of A549 cells compared with doses of 0.78 and 1.56 nM. These results verified that this nanocomposite has the capability to destroy cancer cells by its great photothermal conversion effect. Conversely, under 808 nm laser irradiation, many cancer cells treated with AuNR@S-MCM-41-DOX died, leading to an improved treatment effect than that of AuNR@S-MCM-41-DOX and AuNR@S-MCM-41 + Laser (Fig. 5C). These findings showed that AuNR@S-MCM-41-DOX can quickly release great amounts of DOX through its photothermal conversion action, in addition to being able to induce PTT under 808 nm laser irradiation, leading to the final synergistic chemo-photothermal therapy to A549 cells. In comparison to other in vitro experiments, the cell viability of cells treated with AuNRs@S-MCM-41-DOX + Laser was significantly reduced (35% cell viability) after exposure to near-infrared radiation at a dose of 3.125 nM AuNR@S-MCM-41-DOX and a DOX concentration of 0.0.078 µM. The results were analyzed by two-way ANOVA control groups. To conclude, there is a synergist efficacy in the combination therapy in this nanocomposite; the cell viabilities were compared to the determined value from the additive effect. Particularly, the viability of cells of AuNR@S-MCM-41-DOX (in presence and absence of NIR) and AuNR@S-MCM-41 (in presence of NIR) were examined by MTT assay (Fig. 5D), and results were investigated by two-way ANOVA. The gold nanorods concentration in these three groups is 3.125 nM, and DOX concentration is the same (0.078 µM). After 45 h of incubation with AuNR@S-MCM-41 and AuNR@S-MCM-41-DOX, the cancer cells were exposed to the 808 nm laser and had their media heated to 37, 42, and 47 °C. Furthermore, cells were incubated with AuNR@S-MCM-41-DOX for 48 h, and the viability of cells was 68.2% owing to chemotherapy.

Here, the cell viability of the additive (*f*_additive_) was calculated using the relationship *f*_additive_ = *f*_chemotherapy_ × *f*_PTT_, where *f* is the cell viability for each treatment. It's an additive effect when *f*_combination_ = *f*_additive_, but a synergistic effect occurs when *f*_combination_ is lower than *f*_additive_^53^. The experimental cell viability of the combination treatment (*f*_combination_) (33.1% at 42 °C and 28.12% at 47 °C) was lower than the computed additive interaction of photothermal therapy and chemotherapy (*f*_additive_) (42.98% at 42 °C and 40.1% at 47 °C) of AuNR@S-MCM-41-DOX + Laser, although the results indicated no significant changes in the viability of cells (*p* = 0.38, 0.55). This is expressive of the synergistic anti-cancer efficacy of AuNR@S-MCM-41-DOX + Laser. As shown in Fig. 5D, the increasing therapeutic efficacy of AuNR@S-MCM-41-DOX + Laser was illustrated at temperatures of 42 and 47 compared to 37 °C. The combined chemo-photothermal therapy by AuNR@S-MCM-41-DOX indicated a synergistic effect. The results exhibit that PTT combined with chemotherapy showed an enhanced therapeutic efficacy compared to single chem- or photothermal therapy.

We tested NIR light-triggered DOX release in cells treated with 0.078 µM DOX loaded AuNR@S-MCM-41 using 3.6 W/cm^2^ laser power. This showed 35% viability after NIR exposure, indicating efficacious chemo-photothermal therapy. Compared to prior work using 5 µM DOX and 20 W/cm^2^ laser with Au@SiO_2_, our system provides effective therapy at lower drug doses and laser power^54^.

## Conclusion

In summary, the AuNR@S-MCM-41-DOX nanocomposite was fabricated as a NIR-activated drug delivery structure for combination chemo-photothermal cancer therapy. This new system has good photothermal conversion performance for efficient photothermal therapy. The resulting nanocomposite indicates pH/NIR dual-responsive drug release performances, and the loaded DOX can be released quickly under an acidic pH environment and NIR stimuli. Cellular uptake studies indicate that the AuNR@S-MCM-41-DOX nanocomposite can be efficiently internalized in A549 lung cancer cells and demonstrates good performance in chemo-photothermal combination therapy to kill cancer cells compared to single chemo- or photothermal therapy. The results of this work indicate that this new nanocomposite has good in vitro biocompatibility and great drug loading capacity, making it a promising candidate for combined chemo-photothermal therapy of lung cancer. These promising results will encourage us to further appraise the in vivo anticancer efficiency of the nanocomposite.

### Supplementary Information

Supplementary Information.

## Data Availability

All data generated or analysed during this study are included in this published article and its Supplementary Information files.
