# Supplementary material for: Synergistic chemo-photothermal therapy using gold nanorods supported on thiol-functionalized mesoporous silica for lung cancer treatment
Source: Sci Rep. 2024 Feb 22;14:4373. doi: 10.1038/s41598-024-54778-3 (PMC10884026; doi:10.1038/s41598-024-54778-3)
Supplement: Supplementary file 1 — Supplementary Information. [file 41598_2024_54778_MOESM1_ESM.docx]

**Synergistic Chemo-Photothermal Therapy using Gold Nanorods Supported on Thiol-Functionalized Mesoporous Silica for Lung Cancer Treatment**

Maryam Deinavizadeh^1^, Ali Reza Kiasat*^1,2^, Mohammad Shafiei^3^, Mohammad Sabaeian^4,5^, Roya Mirzajani^1^, Seyed Mohammadsaleh Zahraei^3^, Fateme Khalili^1^, Minmin Shao^6^, Aimin Wu^7^, Pooyan Makvandi*^8,9^, Nasrin Hooshmand*^10^

^1^Department of Chemistry, Faculty of Science, Shahid Chamran University of Ahvaz, Ahvaz, Iran

^2^Petroleum Geology and Geochemistry Research Center (PGGRC), Shahid Chamran University of Ahvaz, Ahvaz, Iran

^3^Department of Biology, Faculty of Science, Shahid Chamran University of Ahvaz, Ahvaz, Iran

^4^Department of Physics, Faculty of Science, Shahid Chamran University of Ahvaz, Ahvaz, Iran

^5^Center for Research on Laser and Plasma, Shahid Chamran University of Ahvaz, Ahvaz, Iran

^6^Department of Otorhinolaryngology, The Second Affiliated Hospital of Shanghai University, Wenzhou Central Hospital, Wenzhou, China

^7^Department of Orthopaedics, Key Laboratory of Structural Malformations in Children of Zhejiang Province, Key Laboratory of Orthopaedics of Zhejiang Province, The Second Affiliated Hospital and Yuying Children's Hospital of Wenzhou Medical University, Wenzhou, 325000, Zhejiang Province, China

^8^Centre of Research Impact and Outcome, Chitkara University, Rajpura, 140401, Punjab, India

^9^Department of Biomaterials, Saveetha Dental College and Hospitals, SIMATS, Saveetha University, Chennai, 600077, India

^10^Laser Dynamics Laboratory, School of Chemistry and Biochemistry, Georgia Institute of

Technology, Atlanta, GA, 30332, USA

**Experimental**

**1. Synthesis of AuNRs**

The AuNRs were synthesized through a two-step process using the seed-mediated growth method^1^. First, gold seeds were prepared by mixing 250 μL of 10 mM HAuCl_4_, 600 μL of 10 mM NaBH_4_, and 7 mL of 100 mM CTAB solution. A color change from yellow to brown was occurred. After the solution was kept at 30 °C for an hour, the growth solution was prepared by mixing 10 mL of 100 mM CTAB, 62.5 μL of 10 mM AgNO_3_, 425 μL of 10 mM HAuCl_4_, and 67.5 μL of 100 mM ascorbic acid. A seed solution of 50 µL was added to the growth solution, then the solution was left overnight at 30 °C. The resulting mixture was then centrifuged twice for 10 min each at 10,000 rpm and washed with deionized water. Finally, the AuNRs were dispersed in 5 mL of water.

**2. Synthesis and modification of MCM-41**

**2.1. Synthesis of mesoporous silica MCM-41**

MCM-41 nanoparticles were synthesized using a surfactant-templated sol-gel method^2^. The process began by heating a mixture of CTAB (1 mmol, 0.4 g) and NaOH (2.54 mmol, 0.102 g) in water at 80°C for 30 min. When the pH reached 12.3, TEOS (8 mmol, 1.7 g) was slowly added and the mixture stirred at 80 °C for 2 h. After cooling, precipitated MCM‐41 was filtrated and washed with refluxing mixture of ethanol /HCl (molar ratio = 100:1) for 6 h at 60°C to extract the surfactant template.

**2.2. Synthesis of mesoporous thiol-functionalized MCM-41, MCM-41-SH**

To grafting of mercatopropyl groups onto the surface of MCM-41, a suspension of MCM-41 (1 g) in anhydrous toluene (100 ml) was mixed with (3-mercatopropyl)trimethoxy-silane (MPTMS) (10 mmol). The resulting mixture was heated under reflux conditions for 24 h under argon protection and then allowed to cool. After filtration, MCM-41-SH was washed with toluene and dried under vacuum for 6 h at 60°C ^3^.

**3. Synthesis of AuNR@S-MCM-41**

A solution of 0.1 wt % MCM-41-SH dispersed in deionized water (140 μL) was added to a stock solution of AuNRs (5 mL). The mixture was stirred at room temperature overnight, and the purified AuNR@S-MCM-41 was obtained through centrifugation two times at 11000 rpm for 10 min to remove the excess MCM-41-SH. Finally, MCM-41-conjugated AuNRs were suspended in the 5 mL of deionized water.

**4. In vitro cellular uptake**

In a 6-well plate, A549 cells (obtained from the Pasture Institute of Iran) were seeded at a density of 100,000 cells per well. After being incubated at 37 °C with 5% CO_2_ for 24 h, the medium was removed and the cells were washed once with PBS. The cells were then exposed to 3 mL dispersion of AuNR@S-MCM-41-DOX in culture media at concentrations of 0.78, 1.56, or 3.125 nM for 48 h. After the exposure period, the cells were washed twice with PBS, trypsinized, and counted. The cells were then centrifuged at 1200 rpm for 5 min, the supernatant was removed, and aqua regia was added to fully digest the cells. The cell-digested solutions were analyzed using ICP-OES after an overnight incubation. The percentage uptake of Au atoms per cell was determined using Equation (1), where C_i_ is the concentration of Au atoms in the incubated dose, C_e_ is the concentration of Au atoms in the cells, N_i_ is the number of cells seeded into each well, and N_e_ is the number of cells after 48 h of treatment.

$$Cell uptake by per cell \left( \% \right)= \frac{\frac{C_{e}}{N_{e}}}{\frac{C_{i}}{Ni}}\times100 (S1)$$

**5. In vitro cytotoxicity**

To evaluate the toxicity of AuNRs conjugates on A549 lung cancer cells (obtained from the Pasture Institute of Iran), MTT assay was employed. The cells were seeded in 96-well plates at a density of 5 × 10^3^ cells/well and incubated in 100 *µ*L of DMEM with 1% FBS for 24 h at 37 °C and 5% CO_2_ to reach confluency. Various concentrations of free DOX (0-100 µM), AuNR@S-MCM-41, and AuNR@S-MCM-41-DOX with equivalent AuNRs concentrations (0-25 nM) were added to the cells, and after 48 h, the supernatant was removed, and 30 µL of MTT solution (0.5 mg/mL) was added into each well. After another 4 h of incubation, the formed formazan crystals were dissolved in 120 µL of DMSO, and the absorbance at 490 nm was measured using a microplate reader (Bio-Tekn Elx 800). For photothermal therapy, cells were incubated for 45 h with AuNR@S-MCM-41 and AuNR@S-MCM-41-DOX at different concentrations with the equivalent AuNRs concentrations before being exposed to 808 NIR laser irradiation (3.6 W/cm^2^) for 5 min. After irradiation, cells were incubated for 2 h, and cytotoxicity was evaluated using MTT assay by equation (2).

$$Cell viability\left( \% \right)=\frac{\left( mean Abs. of treatment group \right)}{\left( mean Abs. of control group \right)}\times100 (S2)$$

**6. Statistical analysis**

All statistical analyzes were conducted using GraphPad Prism v.8.4.3, with experiments being performed in triplicate and repeated three times independently. The t-test, one- and two-way analysis of variance (ANOVA) were used to compare the statistical significance of the cell viabilities under different treatments and concentrations. A probability p-value <0.05 was considered statistically significant in all cases.

References

1 Aioub, M., Panikkanvalappil, S. R. & El-Sayed, M. A. Platinum-coated gold nanorods: efficient reactive oxygen scavengers that prevent oxidative damage toward healthy, untreated cells during plasmonic photothermal therapy. *ACS Nano* **11**, 579-586 (2017).

2 Azaroon, M. & Kiasat, A. R. An efficient and new protocol for the Heck reaction using palladium nanoparticle‐engineered dibenzo‐18‐crown‐6‐ether/MCM‐41 nanocomposite in water. *Appl. Organomet. Chem.* **32**, e4271 (2018).

3 Vrbková, E., Vyskočilová, E. & Červený, L. Functionalized MCM-41 as a catalyst for the aldol condensation of 4-isopropylbenzaldehyde and propanal. *React. Kinet. Mech. Catal.* **114**, 675-684 (2015).
